# Synergistic Enhancement of Carrier Migration by SnO_2_/ZnO@GO Heterojunction for Rapid Degradation of RhB

**DOI:** 10.3390/molecules29040854

**Published:** 2024-02-14

**Authors:** Pengfei Chen, Jin Li, Jianing Wang, Lihan Deng

**Affiliations:** 1 School of Physical Science and Technology, Xinjiang University, Urumqi 830017, China; 107552100728@stu.xju.edu.cn (P.C.); wangjianing@stu.xju.edu.cn (J.W.); denglihan@stu.xju.edu.cn (L.D.); 2 Xinjiang Key Laboratory of Solid State Physics and Devices, Xinjiang University, Urumqi 830017, China

**Keywords:** SnO_2_/ZnO@GO, photocatalysis, quantum dots, rhodamine B, heterojunctions

## Abstract

Organic dyes in natural waters jeopardize human health. Whether semiconductor materials can effectively degrade dyes has become a challenge for scientific research. Based on this, this study rationally prepared different nanocomposites to remove organic dyes effectively. Pure SnO_2_ quantum dots, ZnO nanosheets, and SnO_2_/ZnO (ZS) binary nanocomposites are prepared using the hydrothermal method. Subsequently, SnO_2_/ZnO@GO (ZSG) ternary composites containing different amounts of GO, i.e., ZSG-5, ZSG-15, and ZSG-25, are synthesized by an ultrasonic water bath method, in which ZS was coupled with GO to form Z-type heterojunctions. The ZSG-15 ternary composites exhibited excellent photocatalytic activity for the degradation of rhodamine B by simulating sunlight. The test results show that the degradation rate of ZSG-15 is about 7.6 times higher than ZnO. The increase in photocatalytic activity is attributed to the synergistic effect of SnO_2_ and GO to improve the separation efficiency of photogenerated carriers in ZnO. Notably, the large specific surface area of GO increases the reactive sites. Compared with binary nanocomposites, ZSG-15 broadens the response range to light while further accelerating the electron transport rate and improving the photoelectric stability.

## 1. Introduction

With the rapid development of society in recent years, environmental concerns have grown more prominent, and environmental degradation has become a key challenge confronting humans. The indiscriminate release of industrial, agricultural, and home wastewater has resulted in water contamination, and is therefore of significant concern [1,2]. Water pollution can cause damage to ecosystems as well as jeopardize human health.

Therefore, controlling the emission of pollutants and effective purification of pollutants are essential for environmental protection and ecological improvement [3]. In the search for the effective degradation of pollutants, photocatalytic technology has come to the forefront of scientists’ minds; therefore, the synthesis of photocatalysts for the efficient degradation of pollutants has become a hot topic in today’s research. Photocatalysis is a commonly used method of advanced oxidation that employs semiconductor nanoparticles to break down pollutants using sunlight or artificial light sources emitting ultraviolet or visible light, which helps minimize the production of by-products [4,5]. On the other hand, the photocatalytic oxidation technique has gained popularity owing to its cheap cost and environmental friendliness [6,7,8]. Many metal oxide semiconductors were discovered during the investigation, including ZnO [9], TiO_2_ [10], SnO_2_ [11], Fe_2_O_3_ [12], CuO [13], etc. These semiconductors possess the benefits of being non-toxic, easily prepared, and stable in water, making them extensively used in the early exploratory research phase. Unfortunately, it was found that single-phase metal oxides have some drawbacks, which are prone to photo corrosion phenomenon and low carrier migration efficiency, resulting in a low photocatalytic activity exhibited in degrading organic pollutants [14,15,16]. A variety of methods for effectively solving the problem of the rapid carrier recombination have been identified through exploration, such as the introduction of different atoms for doping, the construction of heterojunctions, the modification of metal oxides by noble metals, and the introduction of carbon-based materials. Interestingly, the formation of heterojunctions not only boosts carrier migration but also diminishes the band gap of metal oxides, improving their photocatalytic activity. A series of binary heterojunctions such as CuO/ZnO heterojunctions [15], TiO_2_/ZnO heterojunctions [17], and SnO_2_@CdS [18] nanowire-quantum dot heterostructures can be synthesized by using preparative methods such as hydrothermal, sol-gel spin-coating, and wet-chemical methods, as demonstrated by previous studies. Compared to single-phase metal oxides, binary heterojunctions hinder the recombination of e-/h+ pairs to some extent, reducing the band gap and improving the utilization of visible light [19]. However, the binary heterojunction still has some defects in the rapid realization of organic dye removal because the transfer rate of electrons is slower, resulting in a lower rate of redox reaction; improving the rate of electron migration is still a significant problem that needs to be solved urgently.

**Scheme 1:**
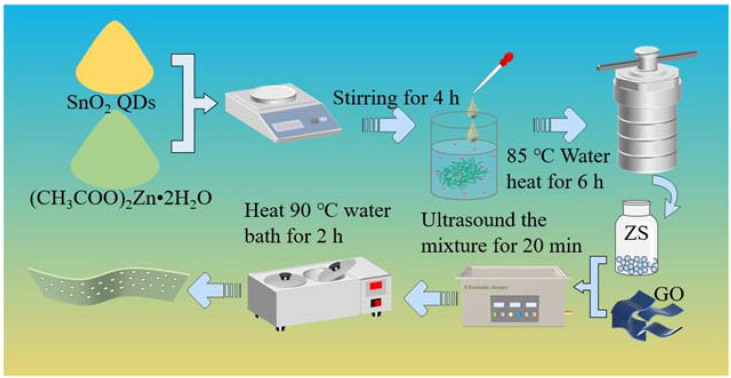
Flow diagram of the SnO_2_/ZnO@GO composites’ preparation process.

Based on this, this experiment introduces GO on the basis of SnO_2_/ZnO to degrade organic dyes rapidly to effectively improve the above problems of binary heterojunction. As we all know, GO has a two-dimensional structure consisting of a single-layer carbon honeycomb network; unique optical, electrical, and thermal conductivity; low density; and high carrier mobility, making it a good modified photocatalytic material that is widely used in catalysis [20,21,22]. Therefore, in this study, a series of binary (ZS) and ternary (ZSG) composites were prepared by hydrothermal and ultrasonic water bath methods (Scheme 1) to compare the adsorption capacity of the dyes as well as the degradation performance, and then the main factors affecting their photocatalytic efficiency were investigated. On the one hand, the high specific surface area of SnO_2_ quantum dots, ZnO nanosheets, and GO nanosheets may supply more reactive active sites and boost the materials’ dye adsorption capability. On the other hand, the tight combination of SnO_2_/ZnO and GO successfully forms a heterojunction, which can effectively improve the carrier mobility and utilization of visible light. In addition, the introduction of GO will lower the bandgap of the material and, at the same time, increase the optical characteristics of ZSG, which causes quicker electron transfer in the photocatalytic degradation process. In photocatalytic experiments, the ZSG ternary composites showed excellent properties. This study provides a novel approach to constructing heterojunctions for semiconductors by investigating the synergistic effects between materials. This introduces an innovative concept to improve the performance of composite materials.

## 2. Results and Discussion

### 2.1. Crystal Structure and Morphology Analysis

**Figure 1:**
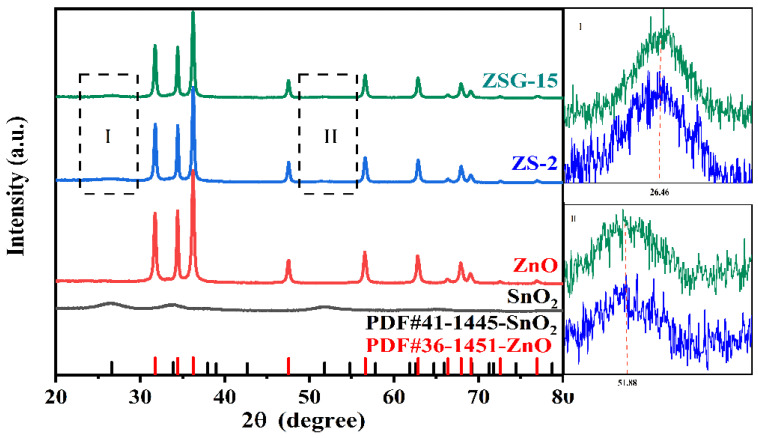
XRD patterns of pure SnO_2_ QDs, ZnO, ZS-2, and ZSG-15 materials.

**Figure 2:**
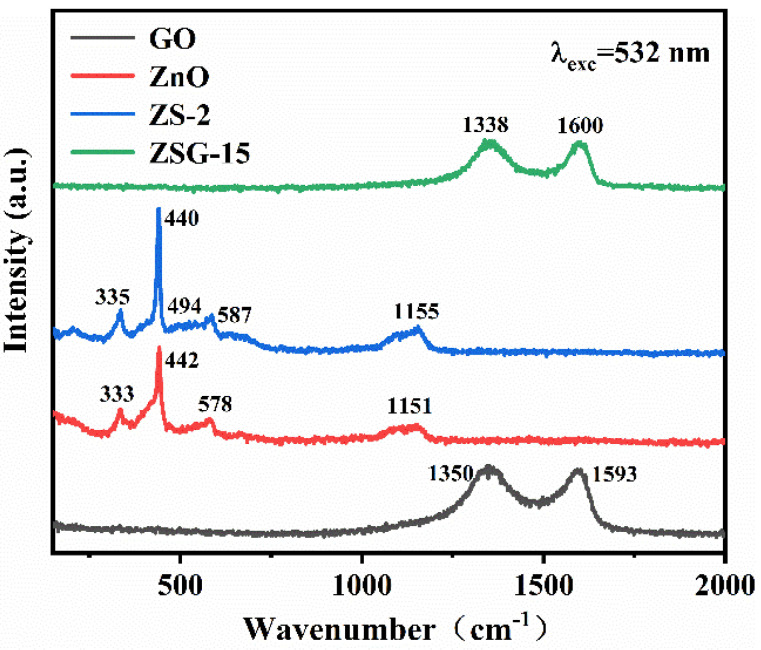
Raman plots of materials ZnO, GO, ZS-2, and ZSG-15.

As shown in Figure 1, the XRD diffraction peaks of ZnO exhibit numerous sharp peaks, suggesting that the obtained ZnO is reasonably well crystallized, and the analysis confirms that the created ZnO agrees with the standard PDF card of ZnO with a hexagonal feldspathic zincite structure (PDF#36-1451). The diffraction peaks occurred at 31.74°, 34.40°, 36.22°, 47.56°, 56.68°, 62.78°, 66.30°, 67.92°, and 69.02°, and correspond to the crystal planes (100), (002), (101), (102), (110), (103), (200), (112), and (201), which is consistent with prior investigations [23]. The SnO_2_ diffraction peaks correlate with the standard PDF card of tetragonal rutile SnO_2_ (PDF#41-1445), and the diffraction peaks are at 26.46°, 33.74°, and 51.88°; their peaks correspond to the SnO_2_ crystallographic planes (110), (101), and (211) [24]. The peak broadening of SnO_2_ diffraction peaks is attributed to its small crystal size and shows the same characteristics in ZS-2 and ZSG-15 [25]. The characteristic diffraction peaks of two components, ZnO and SnO_2_, appeared among the characteristic diffraction peaks of ZS-2 and ZSG-15, respectively. However, due to the small intensity of the diffraction peaks of the SnO_2_ quantum dots and their low content in the composite samples, which resulted in the low intensity of the corresponding peaks in the composite samples, the enlarged diagrams shown in Figure 1 are given. No diffraction peaks of GO were identified in the ZSG-15 composite, which might be attributed to the low concentration and strength of GO [26]. To further demonstrate the presence of GO in the ZSG-15 samples, Raman tests were performed on the ZSG-15 samples, as shown in Figure 2. It is worth noting that no other peaks developed in any of the composites, showing that the ZS-2 and ZSG-15 heterojunctions were successfully constructed.

Figure 2 displays the Raman spectra of GO, ZnO, ZS-2, and ZSG-15, and two distinct vibrational peaks of GO are observed. These peaks correspond to the D peak, generated by the k-phonon A_1g_ symmetric respiration mode, and the G peak, induced by the plane vibration of the E_2g_ phonon of the sp^2^-hybridized carbon atom; the D peak is observed at 1350 cm^−1^, while the G peak is observed at 1593 cm^−1^ [27]. The disordered vibration of graphene at the D peak is used to identify the presence of sp^3^ structural flaws and the placements of graphene edges. The ZSG-15 composite exhibited a blueshift in the D peak and a redshift in the G peak of the GO material. This observation supports the presence of C-O-Zn bonds, suggesting the creation of a heterojunction via the coupling of GO with SnO_2_/ZnO [28,29]. The figure shows that the Raman characteristic peaks of pure phase ZnO are at 333, 442, 578, and 1151 cm^−1^. The Raman bands of the ZnO samples align with the hexagonal structure of zincite. In the ZS-2 composite photocatalysts, there is an additional SnO_2_ characteristic peak at 494 cm^−1^, corresponding to the vibrational mode of E_g_, along with the characteristic peak of ZnO [30]. The matching peak intensity is modest because the SnO_2_ concentration is relatively low, and the ZS-2 Raman spectra demonstrate that the composite material corresponds to two metal oxides, SnO_2_ and ZnO, showing that the composite photocatalyst is constituted of them. It is worth noting that a neutral density filter (ND Filter) was used in the Raman test of the ZSG-15 sample to protect the GO from a burn, focusing on proving the presence of GO in the ZSG-15 sample. As a result, the Raman spectroscopic characterization is critical for the compositional research of the material. After the analysis, the creation of the heterojunction by the SnO_2_/ZnO-coupled GO is highly successful, which is consistent with the results derived from XRD.

**Figure 3:**
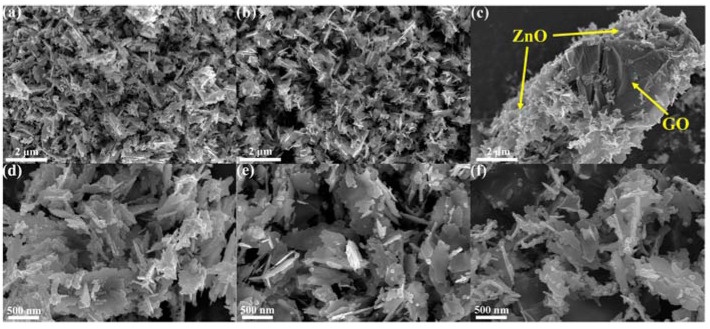
Scanning electron microscope images of the samples of (**a**,**d**) ZnO, (**b**,**e**) ZS-2, and (**c**,**f**) ZSG-15 at 2 µm and 500 nm, respectively.

SEM was used to investigate the microscopic morphologies of pure ZnO, ZS-2, and ZSG-15 photocatalysts, as can be observed in Figure 3a–f. The pure ZnO is a lamellar structure with a lamellar thickness of approximately 14 nm, as illustrated in Figure 3a,d, and the samples are well scattered with no evident aggregation. The Figure 3b,e displays the morphology of ZS-2 samples, it is not easy to discern the unique morphology of SnO_2_ in ZS-2 samples due to the relative size of SnO_2_. However, the ZnO is still in a lamellar structure, indicating that including SnO_2_ quantum dots has essentially no influence on the morphology of ZnO. Interestingly, the microstructure of GO can be observed in Figure 3c,f, where GO has a multilayered lamellar structure. It can also be observed that GO has a large specific surface area, implying a better adsorption capacity for organic dyes, which is consistent with the conclusions drawn from the 30 min dark adsorption of dyes. Furthermore, a substantial quantity of flaky ZnO adhered to the surface of GO is visible in the image, showing that the SnO_2_/ZnO@GO ternary heterojunction was successfully constructed, which is consistent with the findings of XRD and Raman tests.

**Figure 4:**
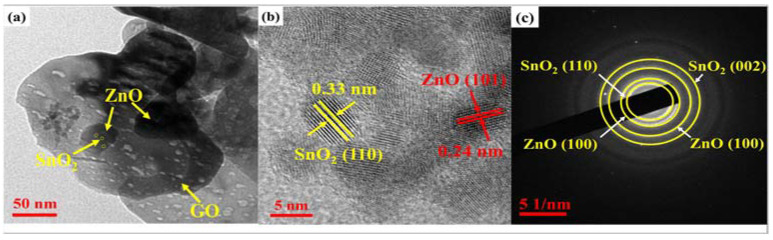
ZSG-15 sample (**a**) TEM morphology; (**b**) HRTEM; (**c**) SAED images.

The microstructure of ZSG-15 nanomaterials was studied using transmission electron microscopy (TEM) and high-resolution transmission electron microscopy (HRTEM) pictures. As shown in Figure 4a, the picture of TEM shows the close adherence of SnO_2_/ZnO to the GO surface in ZSG-15. It was shown that heterojunctions were formed between the binary composites and the GO, which constituted the cladding structure as a whole. This structure enhances the efficient movement of carriers over the interface and photocatalytic efficiency. Figure 4b displays the HRTEM picture of the ZSG-15, and it reveals two distinct lattice fringes, one with a lattice spacing of 0.24 nm and 0.33 nm corresponding to the (101) facet of ZnO and the (110) facet of SnO_2_, respectively, [31] which aligns with the findings derived from XRD. The electron diffraction image in Figure 4c displays distinct diffraction rings that correspond to the crystal planes (110), (002), (100), and (102) of SnO_2_ and ZnO, which indicates that the ZSG-15 is a polycrystalline structure.

### 2.2. Element and Valence Analysis

**Figure 5:**
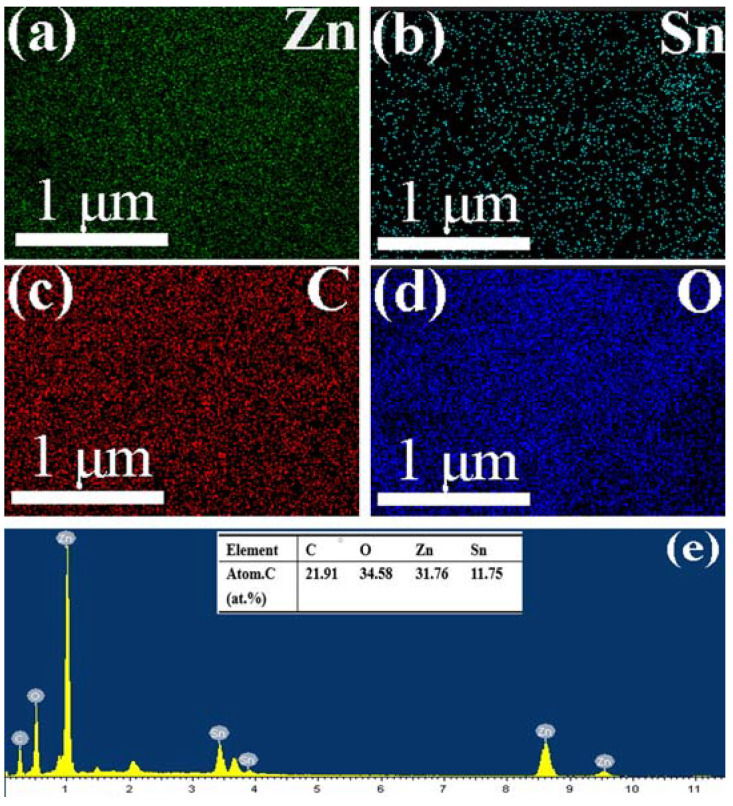
(**a**–**d**) Mapping and (**e**) EDS plots of composite sample ZSG-15 and inset plots of the atomic percentage of different elements.

The elemental composition of the ZSG-15 was confirmed using SEM elemental mapping and EDS plots. Figure 5a–d demonstrates the inclusion of four elements in ZSG-15, Zn, Sn, C, and O, respectively, and those mentioned above may also be observed in Figure 5e, with their respective mass percentages being 31.76%, 11.75%, 21.91%, and 34.58%. Except for element O, element Zn has the highest mass percentage, suggesting that ZnO is the primary constituent of ZSG-15. Furthermore, including C and Sn components is further evidence of the effective formation of the ZSG-15 heterostructure.

**Figure 6:**
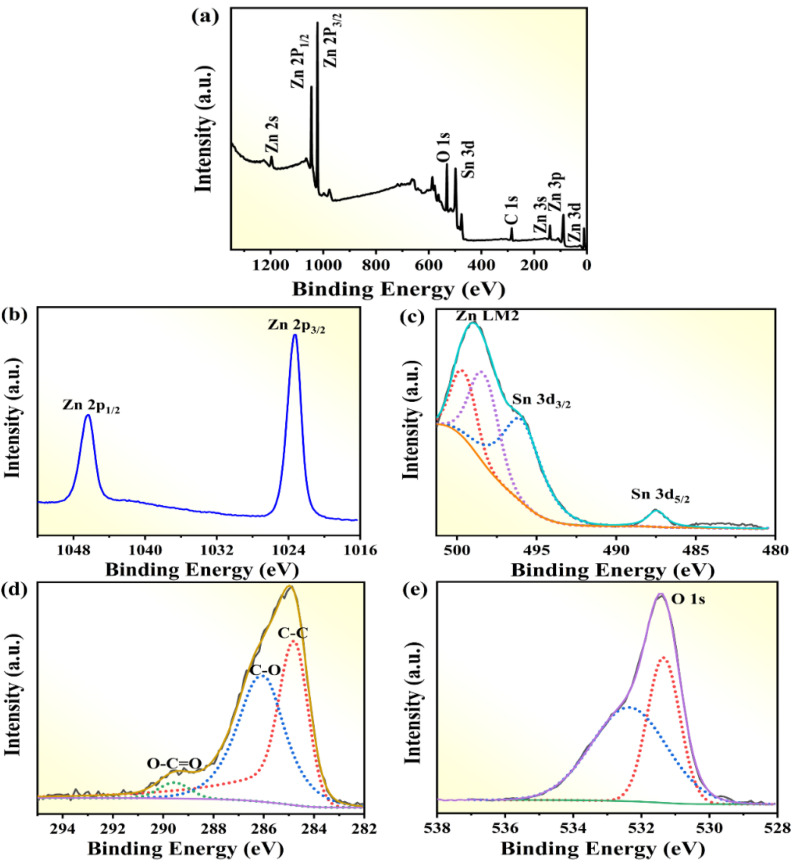
XPS charts of ZSG-15: (**a**) A whole range scan of the bit; (**b**) Zn 2p spectra; (**c**) Sn 3d plots; (**d**) the spectrum of C; (**e**) the spectrum of O 1s.

Figure 6 shows the X-ray photoelectron spectroscopy (XPS) to further examine the chemical composition and chemical valence of the ZSG-15. The different colored curves in the figure represent fits to the valence positions of the chemical elements. The results in Figure 6a indicate that the ZSG-15 mainly comprises four elements: Zn, Sn, O, and C. According to Figure 6b, the presence of Zn 2p_1/2_ and Zn 2p_2/3_ peaks have binding energies of 1021.1 eV and 1044.9 eV, respectively, with a peak separation of 23.8 eV, indicating that Zn’s valence is +2 [32,33]. The prominent peaks in Figure 6c correspond to Sn 3d_5/2_ and Sn 3d_3/2_, respectively, indicating that the Sn ions in SnO_2_ are +4 valent, and the difference in the binding energy of SnO_2_ is 8.4 eV, which is consistent with previously reported results in the literature [34]. Notably, the peak of Sn 3d_3/2_ coincides with the Zn LM2 helix, and comparable results have been reported in other papers [35,36]. Figure 6d exhibits the XPS peaks of C 1s in the ZSG-15, 284.8 eV corresponds to C-C bonds, 286 eV to C-O bonds, and 289.5 eV to O-C=O bonds [37,38]. As can be observed in Figure 6e, the asymmetric distribution of O 1s can be fitted to two symmetric peaks at 531.35 and 532.36 eV, indicating two distinct forms of lattice oxygens. The peak at 531.35 eV corresponds to the O^2−^ ions due to the Zn-O bond in ZnO, and the peak at 532.36 eV is attributed to the OH groups on the surface of ZSG-15 [39,40].

### 2.3. Optical Properties Analysis

**Figure 7:**
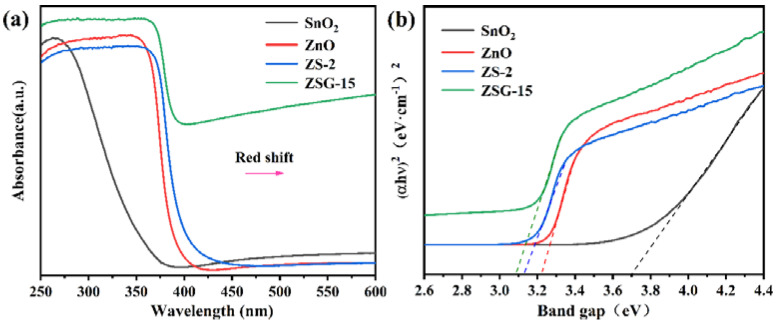
(**a**) Light-scattering patterns of SnO_2_, ZnO, ZS-2, and ZSG-15 samples in the UV-Vis range; (**b**) Plots of the band gaps of SnO_2_, ZnO, ZS-2, and ZSG-15 samples.

Figure 7a displays the UV-Vis diffuse reflectance spectra (UV-Vis DRS) of the different samples synthesized, from which we can observe that the corresponding curve patterns of the different samples are approximately the same. With the incorporation of GO, the absorbance increases immensely; there is a significant redshift of the ZSG-15 absorption boundary compared with ZnO and ZS-2, which was around 420 nm. ZSG-15 broadens the light response range and improves the utilization of visible light. Hence, the incorporation of GO into ZSG-15 enhances the degradation of organic pollutants. Furthermore, the band gaps of different semiconductor materials have been calculated using the Kubelka–Munk function: (αhv)^2^ = A (hv − E_g_), where α represents the absorption coefficient, h denotes Planck’s constant, v signifies the frequency of the light, E_g_ represents the optical bandgap, and A is the constant of the sample, as illustrated in Figure 7b [41]. The results indicate that the band gaps of SnO_2_, ZnO, ZS-2, and ZSG-15 are 3.70 eV, 3.23 eV, 3.11 eV, and 3.08 eV, respectively. The decrease in the band gap of the ZSG-15 confirms the successful combination of GO with the ZS to create a heterojunction. This finding aligns with the outcomes of the Raman test and SEM characterization.

**Figure 8:**
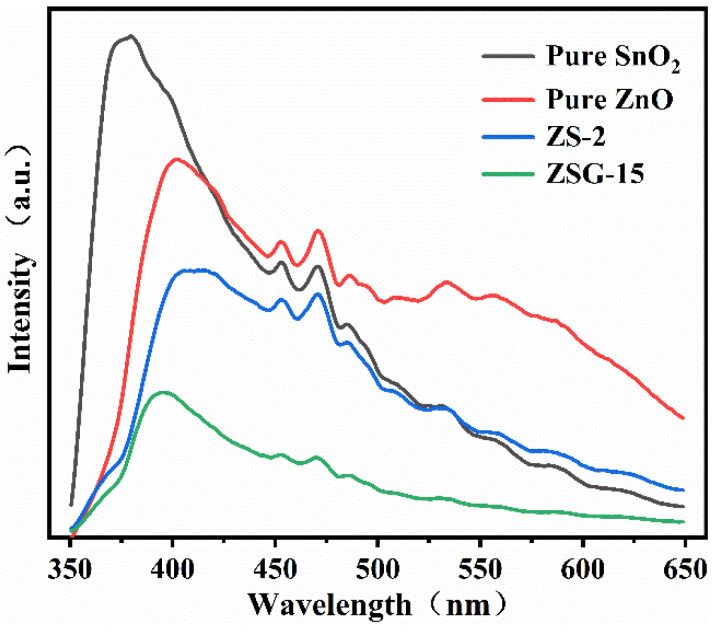
PL spectra of samples SnO_2_, ZnO, ZS-2, and ZSG-15.

Figure 8 shows the PL spectra of pure SnO_2_, ZnO, ZS-2, and ZSG-15 in the range of 350–650 nm. The result illustrates that the emission peak intensity is highest for pure SnO_2_ and lowest for ZSG-15, and the photogenerated electron-hole pair mobility is inversely proportional to the PL intensity, with lower intensities implying a better carrier mobility efficiency [42]. The photoluminescence intensity of GO tends to 0 through the experiments of S et al. Thus, the introduction of GO makes the photoluminescence intensity of ZSG-15 lower than the ZS-2 material [43]. Therefore, the mobility of electrons and holes in ZSG-15 is the largest, indicating that the heterojunction of ZSG-15 efficiently suppresses electron and hole recombination. Effective charge separation may boost sample longevity and interfacial charge transfer efficiency, enhancing photocatalytic activity, which suggests that the ZSG-15 samples have higher levels of active photocatalytic activity [44]. The PL results confirm that the heterojunction formation of the ZSG-15 photocatalyst reduces the free carrier complexation and thus improves the photocatalytic activity of the samples, consistent with the photocatalytic degradation experiments of RhB organic pollutants.

### 2.4. Electrochemical Properties

**Figure 9:**
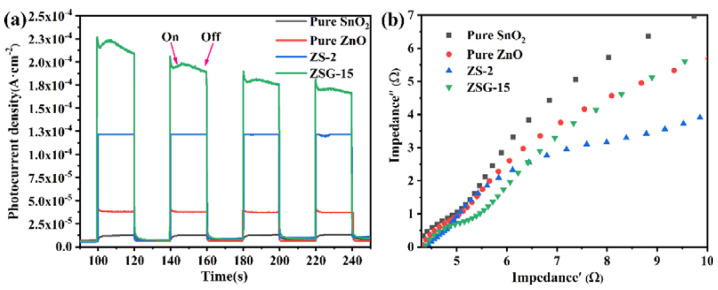
(**a**) Transient photocurrent response plots of SnO_2_, ZnO, ZS-2, and ZSG-15 samples; (**b**) EIS Nyquist plots of SnO_2_, ZnO, ZS-2, and ZSG-15 samples.

The transient photocurrent response (TPR) and electrochemical impedance spectroscopy (EIS) of various materials were examined by electrochemical techniques to verify the free carrier movement. As shown in Figure 9a, the switching time interval of the photocurrent test is 20 s, and the photocurrent densities of all the samples are zero when no light is applied to the samples. However, once the light source is turned on to irradiate the samples, the corresponding samples’ photocurrent densities increase rapidly, suggesting that the samples exhibit a high response speed during the photocurrent test. The significant rise in the photocurrent density may be ascribed to the fast movement of charges at the interface between the sample applied on the conducting glass sheet and the electrolyte. Meanwhile, it can be observed that the presence of GO increases the magnitude of the photocurrent density, which is the largest for ZSG-15. It is attributed to the fact that the SnO_2_ quantum dots and ZnO nanosheets are tightly encapsulated in the lamellar structure of GO, which leads to the fast diffusion transport of carriers. The introduction of GO has been shown to suppress the recombination of free carriers efficiently, and this finding further supports the notion that the formation of a heterojunction may successfully impede the combination of photogenerated electrons and holes, aligning with the conclusion drawn from PL. The Nyquist plots of the prepared samples are shown in Figure 9b, and the charge separation effectiveness of the produced samples was further examined by analyzing the semicircle in the frequency area of the Nyquist plot [45]. Notably, the ZSG-15 impedance curves exhibit a semicircular form and have the shortest radius, suggesting a reduced impedance; it is advantageous for enhancing the interfacial charge transfer. The electron uptake and transport characteristics of GO in the composites facilitate the separation of carriers, leading to enhanced photocatalytic rates. The preceding findings confirm that the ZSG-15 exhibits a high degree of separation efficiency and exceptional photocatalytic activity in the microphysical process of carrier separation, which aligns with the analysis of its photocatalytic activity.

### 2.5. Photocatalytic Activity Evaluation

**Figure 10:**
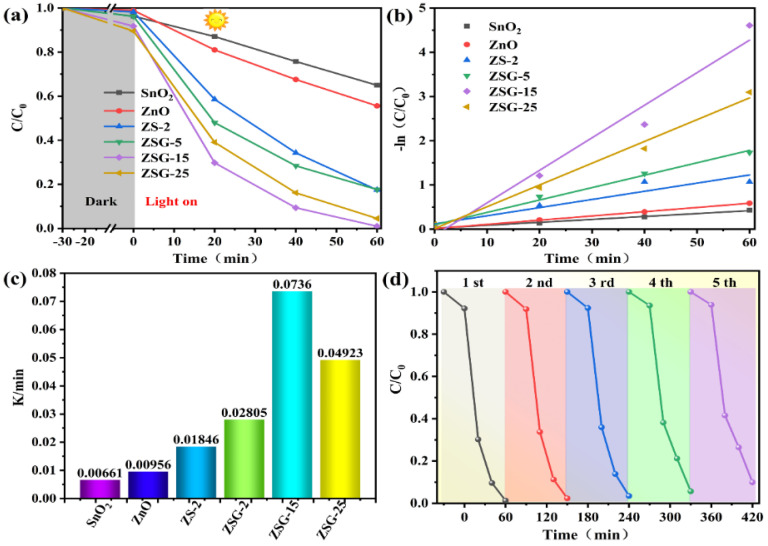
(**a**) Photocatalytic degradation efficiency curves of SnO_2_, ZnO, ZS-2, and ZSG-series samples; (**b**) first-order kinetic curve fit for photocatalytic degradation of all the samples; (**c**) K-value histograms of all the samples; (**d**) cyclic test plots of ZSG-15 samples.

The photocatalytic activity of the SnO_2_, ZnO, ZS series, and ZSG series nanocomposites was examined by measuring the degradation of RhB as a pollutant, and the result can be observed in Figure 10a. In the photocatalytic studies, 10 mg of various samples were consecutively measured and introduced into a 50 mL solution contaminated with RhB at a concentration of 15 mg/L, and the catalytic reaction was then conducted under a xenon lamp. First, before the catalytic reaction, a dark reaction was carried out on all samples for 30 min to help the mixture reach the adsorption-desorption equilibrium. Then, the xenon lamp was switched on to conduct the photocatalytic reaction for 60 min. Based on the data shown in Figure 10a, it was observed that the adsorption capacity of the samples increased with the addition of the additional GO, which is attributed to the significant rise in the specific surface area of GO. The photodegradation curves of RhB solutions for all samples were also determined using Equation (1) [46].

(1) D%=1−CC0×100%

After 60 min of a photocatalytic reaction, the degradation efficiencies of SnO_2_, ZnO, ZS-2, ZSG-5, ZSG-15, and ZSG-25 on the RhB solution were 32.5%, 43.5%, 82.2%, 82.7%, 98.9%, and 94.9%, respectively. The high catalytic activity of ZSG-15 may be ascribed to its strong responsiveness to the visible light and efficient production of electron-hole pairs upon excitation by visible light. The superior photocatalytic performance of ZSG-15 compared to ZS-2 demonstrates the synergistic influence of the three constituents. Figure 10b displays the natural logarithm of the ratio of *C* to *C_0_* plotted against the duration of exposure to light [47].

(2) −ln⁡CC0=Kt

where *t* represents the time, and *C*_0_ and *C* represent the initial and co-corresponding moments of RhB concentration of the contaminated liquid, respectively. The photocatalytic degradation first-order kinetic curves of all the samples and the degradation rate (K-value) histograms of the samples in Figure 10b,c show that the degradation rate of the ZSG-15 was the highest, which is 7.6 and 3.9 times higher than that of pure ZnO and ZS-2, respectively, indicating that the formation of the heterojunction by the coating of SnO_2_/ZnO@GO and the synergistic action of the three components can effectively improve the photocatalytic efficiency. The literature results regarding the percentage degradation of organic pollutants by various photocatalysts incorporating GO and SnO_2_/ZnO binary composites are summarized in Table 1. In addition to testing the catalytic activity of ZSG-15, we also evaluated its catalytic stability. As illustrated in Figure 10d, five cyclic degradation experiments were performed on the samples, and the catalytic activity of the samples decreased due to the loss of the samples during recycling. The catalytic efficiency achieved on the fifth attempt was 87%, suggesting that the sample retains a significant amount of catalytic activity and had excellent cyclic stability.

### 2.6. Possible Photocatalytic Mechanism Investigation

**Figure 11:**
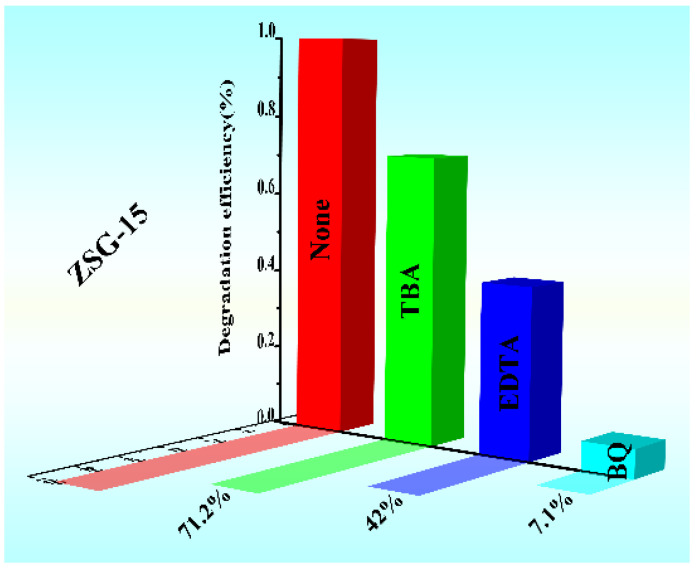
Histogram showing how the addition of TBA, EDTA, and BQ radical trappers affected the rate at which RhB degraded in ZSG-15 samples.

As shown in Figure 11, free radical trapping experiments were conducted on ZSG-15 to identify the reactive chemicals generated throughout the reaction process and examine the reaction mechanism in detail [53,54]. During the capture experiments, the photocatalyst is exposed to light, causing it to produce electron-hole pairs, and the electrons and holes then interact with adsorbed oxygen and water on the photocatalyst’s surface. This interaction leads to the formation of different free radicals, which are crucial in the degradation of organic dye molecules. Tert-Butyl Alcohol (TBA, 5 mM), benzoquinone (BQ, 5 mM), and disodium ethylenediaminetetraacetic acid (Na_2_-EDTA, 5 mM) were selected as the capturing agents for hydroxyl radicals (•OH), superoxide radicals (•O_2_^−^), and holes (h^+^) in this experiment. Upon adding 5 mL of BQ, the photocatalytic degradation efficiency of RhB organic pollutants by ZSG-15 at 60 min was 7.1%. However, the addition of the trapping agent significantly suppressed the removal of RhB by the ZSG-15, which suggests that •O_2_^−^ is the primary active substance in the reaction system; this plays a crucial role in the degradation of RhB. Furthermore, the photocatalytic degradation efficiency of the ZSG-15 for RhB organic pollutants was only 42% at 60 min when 5 mL EDTA was added, and the observation suggests that h+ is an important active ingredient in the reaction system. Adding 5 mL of TBA did not significantly impact the photocatalytic activity of the ZSG-15. The efficiency of the ZSG-15 in degrading RhB organic pollutants after 60 min was 71.2%, which suggests that •OH has a secondary function in the reaction system.

**Figure 12:**
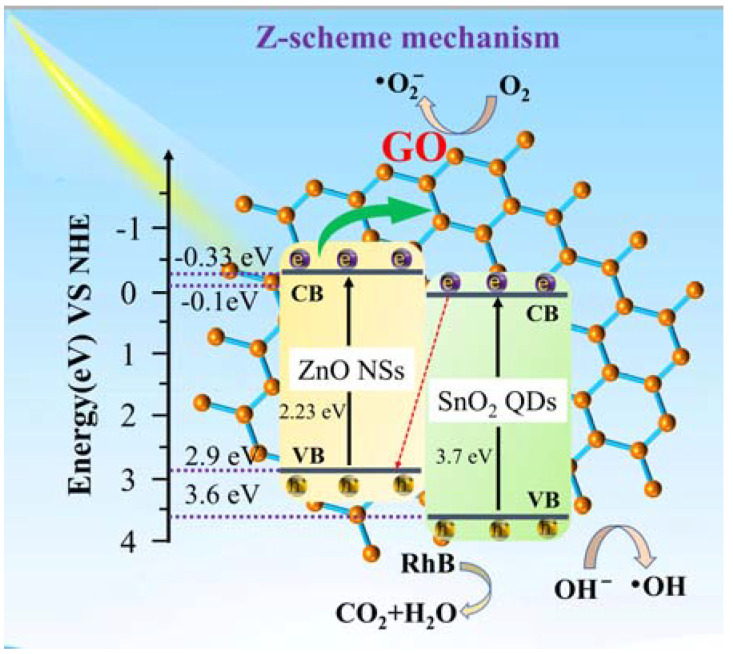
Potential process for ZSG materials’ photocatalytic destruction of organic contaminants.

The photocatalytic degradation process of ZSG composites was hypothesized based on the results of free radical quenching studies, as observed in Figure 12. The diagram illustrates the process of the RhB solution degradation by ZSG photocatalysts, which may be categorized into three stages: (I) creation of e^−^ and h^+^, (II) generation of active species, and (III) degradation of RhB molecules. The empirical equations determine the energy band locations of SnO_2_ and ZnO semiconductors [23,55].

(3) $$E_{VB}=\chi -E_{e}+0.5E_{g}$$

(4) $$E_{CB}=E_{VB}-E_{g}$$

The widths of the valence band, conduction band, and band gap are denoted as *E_VB_*, *E_CB_*, and *E_g_*, respectively. At the atomic scale of hydrogen, the magnitude of its free electrons and the electrical conductivity of the substance are denoted by *Ee* and *χ*, respectively; the value of *E_e_* is 4.5 eV, and the value of *χ* is 6.25 and 5.79 eV for SnO_2_ and ZnO, respectively [56,57]. The energy bandgaps of SnO_2_ and ZnO, as shown in Figure 7b, are determined to be 3.7 eV and 3.23 eV, respectively. Consequently, the valence band potentials of SnO_2_ and ZnO are calculated to be 3.6 eV and 2.9 eV, respectively, based on the empirical equations. Meanwhile, the corresponding conduction band potentials were calculated to be −0.1 eV and −0.33 eV, respectively. If the SnO_2_/ZnO exhibits a type-II heterojunction, the photogenerated electrons will be efficiently moved from the conduction band of ZnO to the conduction band of SnO_2_, and at the same time, the photogenerated holes on the valence band of SnO_2_ will be effectively transferred to the valence band of ZnO. If the e^−^/h^+^ satisfies the above transfer case, the photogenerated electron is located in the conduction band of tin dioxide and the photogenerated hole is located in the valence band of zinc oxide. Because the conduction band potential of SnO_2_ is closer to a positive value than the standard redox potential of O_2_/•O_2_^−^ (O_2_/•O_2_^−^, −0.33 eV vs. NHE) [58], the intermediate reactive radical •O_2_^−^ cannot be generated; however, because •O_2_^−^ is the most critical active substance in the degradation process, the mechanism of SnO_2_/ZnO is not compatible with a type II heterojunction. As a result, the electron transfer in SnO_2_/ZnO is more consistent with direct Z-type heterojunctions, in which electrons in the conduction band of SnO_2_ with a low reduction capacity are transferred to the valence band of ZnO under light conditions and e^−^/h^+^ complexation occurs concurrently. At the same time, the electrons on the ZnO conduction band were rapidly captured by GO nanosheets, and the electrons on the GO nanosheets reacted with O_2_ on the photocatalysts’ surfaces to generate •O_2_^−^ active substances. The addition of GO nanosheets improved the optical properties of the ZSG composites, which was the reason for the faster electron transfer during the photocatalytic degradation process. Furthermore, the holes in the valence band of SnO_2_ engage in the oxidation process, oxidizing the RhB molecule to CO_2_ and H_2_O, which is consistent with the findings of the previous sacrificial agent tests. As a result of the above logical analysis, the Z-type heterojunction with GO co-catalyst may effectively block the complexation of photogenerated carriers, resulting in more strong redox characteristics and an improved photocatalytic degradation performance. The following reactions are involved in the photocatalytic degradation process [5,59]:

ZnO − SnO_2_/GO + h*v*→ZnO − SnO_2_(h^+^) + GO(e^−^) (5)

ZnO − SnO_2_(h^+^) + H_2_O→ZnO − SnO_2_ + •OH + H^+^ (6)

h^+^ + OH^−^→•OH (7)

GO(e^−^) + O_2_→GO + •O_2_^−^ (8)

•O_2_^−^ + H_2_O→HO_2_• + OH^−^ (9)

GO(e^−^) + HO_2_• + h^+^→H_2_O_2_ + GO (10)

GO(e^−^) + H_2_O_2_ + h^+^→•OH + H_2_O + GO (11)

RhB + •OH→CO_2_ + H_2_O (12)

RhB + •O_2_^−^→CO_2_ + H_2_O (13)

## 3. Materials and Methods

### 3.1. Materials

Zn(CH_3_COOH)_2_•2H_2_O, SnCl_4_•5H_2_O, and graphite flakes were obtained from Aladdin Bio-Reagent Co. for use in this study. Sodium hydroxide (NaOH), urea (CO(NH_2_)_2_), and cetyltrimethylammonium bromide (CTAB) were provided by McLean Bio-Reagent Co. Unless otherwise noted, all commercial compounds used in the studies were of analytical quality and did not need extra purification. The solvents used in the studies were only deionized water.

### 3.2. Synthesis of SnO_2_ Quantum Dots

We measured the mass of 1.75 g of SnCl_4_•5H_2_O and 0.6 g of CO(NH_2_)_2_. Two compounds were dissolved in 30 mL of deionized water and agitated for 10 min. The solution, which included CO(NH_2_)_2_, was then added gradually to the solution of SnCl_4_•5H_2_O to create a combined solution, and the mixture was swirled for 30 min. The mixture was placed into a 100 mL reactor and hydrothermally heated for 2 h at 180 °C. After cooling to an ambient temperature, the products were centrifuged five times with deionized water and anhydrous ethanol before being dried at 80 °C for 10 h.

### 3.3. Sample Preparation for GO, ZS, and ZSG

As previously documented in the literature, Hummer’s technique was used to produce GO [60]. The SnO_2_/ZnO (ZS) composite samples were produced in the following manner: 12 mg, 24 mg, and 36 mg of SnO_2_ were weighed and added to 20 mL of deionized water, accordingly, while 1.4 g of CTAB was added to the aqueous SnO_2_-filled solution and agitated for 4 h. The agitated mixture was added to a mixed solution of 3 mmol of Zn(CH_3_COOH)_2_•2H_2_O and 10 mmol of NaOH and stirred for 10 min before being transferred to a 100 mL reactor and hydrothermalized at 85 °C for 6 h before being centrifuged and dried. Finally, the resultant white powder was transported to a muffle furnace for 2 h of calcination at 600 °C. Based on the inclusion of varying mass ratios of SnO_2_ powder, the SnO_2_/ZnO composite samples were called ZS-1, ZS-2, and ZS-3.

The SnO_2_/ZnO@GO (ZSG) composite samples were synthesized as follows: the production technique for the ternary complex ZSG was similar to that for the binary complex, except that varying amounts of the GO suspension were added. Different volumes of prepared GO materials (5 mg, 15 mg, and 25 mg) were added to 10 mL of deionized water and sonicated for 10 h to generate a wholly disseminated GO solution. The dispersed GO suspension was added to the ZS-2 suspension, which was then sonicated for 30 min before being put in a water bath and agitated at 90 °C for 2 h. The resulting SnO_2_/ZnO@GO heterostructures were designated as ZSG-5, ZSG-15, and ZSG-25 in that sequence. The Scheme 1 illustrates the procedure for creating ZSG composites using micro and nano ZSG. The hydrothermal method is used to produce SnO_2_ quantum dots by hydrolyzing urethane as a base source, resulting in the release of CO_2_ and NH_3_ when heated to 180 °C. The interaction between NH_3_ and H_2_O produces NH_3_•H_2_O. Ammonia then decomposes and combines with tin salts to make Sn(OH)_6_^2−^, which eventually leads to the creation of SnO_2_ as shown in reactions (14, 15, 16, and 17) [61]. While manufacturing ZnO, the zinc salt reacts with OH^−^ to produce Zn(OH)_4_^2−^, which ultimately leads to the formation of ZnO, as observed in reactions (18, 19) [62].

CO(NH_2_)_2_ + 3H_2_O→2NH_3_•H_2_O + CO_2_ (14)

NH_3_•H_2_O→NH_4_^+^ + OH^−^ (15)

Sn^4+^ + 6OH^−^→Sn(OH)_6_^2−^ (16)

Sn(OH)_6_^2−^→SnO_2_ + 2H_2_O + 2OH^−^ (17)

Zn^2+^ + 4OH^−^→Zn(OH)_4_^2−^ (18)

Zn(OH)_4_^2−^→ZnO + H_2_O + 2OH^−^ (19)

### 3.4. Photocatalyst Characterization

To produce X-ray diffraction spectra, the physical phase analysis of the different materials was performed using an X-ray diffractometer (XRD, Bruker D8 Advance, Ettlingen, Germany) with Cu k*α*1 (*λ* = 0.154 nm) radiation. A LabRAM HR evol spectrometer with a 532 nm excitation wavelength was used to obtain the Raman spectra, and neutral-density filters were also used (ND Filters). The samples’ elemental distribution and surface micromorphology were also evaluated using a field emission scanning electron microscopy (SEM, LEO1430VP, Oberkochen, Germany). The fine morphology of the samples and information on the crystal structure was determined using a transmission electron microscopy (TEM, JEM-2100, Tokyo, Japan) and high-resolution transmission electron microscopy, respectively. Each sample’s UV-Vis diffuse reflectance spectra were acquired using a UV-Vis spectrophotometer (UV-Vis, Hitachi U-3010, Tokyo, Japan) calibrated with BaSO_4_. A fluorescence spectrophotometer (F4600, Hitachi, Shimadzu, Japan) with an excitation wavelength of 325 nm was used to measure photoluminescence (PL) spectra. Relevant data from the X-ray photoelectron spectroscopy (XPS, Thermo ESCALAB 250XI, Newington, NH, USA) were collected to assess the elemental composition and valence of the materials.

### 3.5. Determination of Photocatalytic Activity

A 300 W xenon lamp (PLS-SXE300, Perfectlight Technology Co., Beijing, China) was used to catalyze the reaction of rhodamine B (15 mg/L). The reaction protocol was weighing 10 mg of pure ZnO, SnO_2_, ZS series, and ZSG series powders into 50 mL (50 mL of the mixture was taken from a volumetric flask containing 1 L of deionized water and 15 mg of RhB) of 15 mg/L RhB liquid and ultrasonically dispersing them for 10 min at room temperature. Second, a dark reaction was to be performed before turning on the light source pair, i.e., stirring for 30 min without turning on the light source pair to bring the solution to an adsorption–desorption equilibrium. Finally, the xenon light was switched on for 60 min of photocatalytic activity. After the degradation was completed, the combined solution was centrifuged, and the UV-Vis spectra of the liquids at various time intervals were collected using a UV-Vis spectrophotometer.

## 4. Conclusions

SnO_2_/ZnO@GO heterojunction photocatalysts were created using a simple hydrothermal approach and an ultrasonic water bath method. Combining GO in various mass ratios created three composite samples: ZSG-5, ZSG-15, and ZSG-25. SnO_2_ and GO considerably improved the photocatalytic performance of ZnO in this research. To identify the key factors that promote ZnO photocatalytic degradation, the material was thoroughly evaluated using XRD, Raman, SEM, TEM, UV-Vis, XPS, PL, and other methods in terms of physical, structural, morphological, and optical characteristics. When the light exposure period reached 60 min, the degradation efficiency of RhB by the ZSG-15 was close to 99%, and the composite material exhibited a superior catalytic activity than the single material. It was shown that creating heterojunctions improved the photocatalytic activity of the materials. The ZSG-15 samples were also exposed to five cycle studies, with the degradation efficiency of RhB reaching 87% for the fifth time, indicating the ZSG-15 composite samples’ stability and reusability. The composites’ vigorous catalytic activity was ascribed to the fact that SnO_2_ quantum dots, ZnO nanosheets, and GO nanosheets all had large specific surface areas, which can supply a large number of active sites. The SnO_2_/ZnO@GO heterojunction enhanced carrier separation efficiency while widening the photoresponse range. Meanwhile, using free radical quenching experiments, this paper proposed that the catalytic mechanism of the SnO_2_/ZnO@GO heterojunction was consistent with the direct Z-type heterojunction, which was designed to inhibit a e^−^/h^+^ recombination while accelerating the carrier transfer and improving photocatalytic activity. This study aims to provide an effective strategy for the development of photocatalysts with innovative heterojunctions, offering a novel idea for the rapid degradation of organic pollutants.

## Figures and Tables

**Table 1:** Comparing the photocatalytic activity of several semiconductor nanomaterials (SnO_2_/ZnO) and GO-modified materials published in the literature.

S.NO	Photocatalyst	Preparation Method	Pollutant	Degradation Efficiency	Ref.
1	NiFe2O4@GO	Hydrothermal	MB	100% in 120 min	[48]
2	CdSe/GO	Aqueous phase	BG	95% in 90 min	[49]
3	Ag/GO/TiO2	Aqueous phase	MB	97% in 180 min	[50]
4	ZnO@SnO2	Hydrothermal	TC	91.2% in 90 min	[51]
5	ZnO/GO	Solid phase	CV	99% in 240 min	[52]
6	SnO2/ZnO@GO	Aqueous phase	RhB	98.9% in 60 min	This work

## Data Availability

The data presented in this study are available on request from the corresponding author.
